# Assessing the Structure of the Five Factor Model of Personality (IPIP-NEO-120) in the Public Domain

**DOI:** 10.5964/ejop.v15i2.1671

**Published:** 2019-06-07

**Authors:** Petri J. Kajonius, John A. Johnson

**Affiliations:** a Department of Psychology, University of Gothenburg, Gothenburg, Sweden; b Department of Psychology, University West, Trollhättan, Sweden; c Department of Cognitive Neuroscience and Philosophy, University of Skövde, Skövde, Sweden; d Department of Psychology, Pennsylvania State University, State College, USA; Webster University Geneva, Geneva, Switzerland

**Keywords:** personality, structure, Five Factor Model, IPIP

## Abstract

Assessment of individual differences in personality traits is arguably one of the hallmarks of psychological research. Testing the structural validity of trait measurements is paramount in this endeavor. In the current study, we investigated 30 facet traits in one of the accessible and comprehensive public-domain Five Factor Model (FFM) personality inventories, IPIP-NEO-120 (Johnson, 2014), using one of the largest US samples to date (*N* = 320,128). We present structural loadings for all trait facets organized into respective FFM-trait domain (Neuroticism, Extraversion, Openness, Agreeableness, and Conscientiousness). Both hierarchical second-order and bi-factor models showed tolerable model fit indices, using confirmatory factor analysis in a structural equation modeling (SEM) framework. Some facet traits were substantially more representative than others for their respective trait domain, which facilitate further discussions on FFM-construct content. We conclude that IPIP-NEO is sufficiently structurally robust for future use, for the benefit of research and practice in personality assessment.

Understanding how measurements of individual differences are structured is one of the foundations of psychology. A particular interest for assessment of personality traits has for some time been on the rise, even in fields outside psychology (Pennisi, 2016; Swann & Seyle, 2005). The arguably most used conceptualization for personality is the Five Factor Model (FFM; Costa & McCrae, 1995; McCrae, 2010), consisting of trait domains Neuroticism, Extraversion, Openness, Agreeableness, and Conscientiousness. However, underlying the five trait domains is a number of specific lower order facet traits that makes up a more fine-grained structure, which hitherto has been less emphasized. Recently, editors have called for increased attention to facet trait analysis as a way forward for research in personality testing (Ziegler & Bäckström, 2016). The present study sought to advance this call by testing one of the more accessible and comprehensive public-domain representations of the FFM, IPIP-NEO-120 (Johnson, 2014). The IPIP-NEO is a product of the International Personality Item Pool collaboration project (IPIP; Goldberg et al., 2006). Both researchers and practitioners have free access to this instrument, and to the authors’ understanding it is frequently used in behavioral sciences across Europe. The IPIP-NEO has to our knowledge only been subjected to structural analyses in small samples (e.g., Kajonius & Dåderman, 2014) or in short versions (e.g., mini-IPIP; Kajonius & Dåderman, 2017). In the present study we were able to secure 30 facet traits based on 120 personality items in a large public US sample (*N* = 320,128).

Without a psychometrically sound understanding of an open-source FFM instrument such as the IPIP-NEO-120, implications concerning use and interpretation may be skewed or erroneous (see Mõttus, 2016). Not knowing how facet traits organize and contribute to the FFM we may not know what the five trait domains imply in terms of scope, precision, and consequently not knowing what the FFM measures. These investigations may help inform on the theoretical and practical use of personality measurement in the public domain.

## The Five Trait Domains of Personality

Personality traits are important for most, if not all, life outcomes, and have demonstrated predictive validity in subjective outcomes such as relationships and well-being (Roberts, Kuncel, Shiner, Caspi, & Goldberg, 2007). Traits also relate to a variety of objective life-outcomes, such as annual income and educational attainment, as shown in studies with a Swedish nation-wide sample (e.g., Kajonius & Carlander, 2017). Personality traits furthermore seem to be growing in importance with the contexts of individualism in modern society (Skirbekk & Blekesaune, 2014). There is also emerging evidence that personality traits tend to cluster geographically (Jokela, Bleidorn, Lamb, Gosling, & Rentfrow, 2015; Kajonius & Mac Giolla, 2017), providing nations, regions, or neighborhoods with certain characteristics. Personality traits are principally known to be fairly stable and develop predictably throughout life (Briley & Tucker-Drob, 2014). A study based on almost 15 million twin pairs (Polderman et al., 2015) reported that the heritability for personality traits generally is 50% (*h^2^* = .49). The majority of reliable (error-corrected) trait variance over a life-time (83%) has been shown to be due to stable influences such as genetics and social maturation (Anusic & Schimmack, 2016).

Personality is most frequently measured with the five factor model (FFM; McCrae, 2010). This represents regularities of thoughts, feelings, and behaviors in individuals expressed in five broad trait domains: N = Neuroticism, E = Extraversion, O = Openness, A = Agreeableness, C = Conscientiousness. Recurring correlations among these have reignited the importance of studying the structure of the FFM (Strus, Cieciuch, & Rowiński, 2014). This has particularly come to the forefront with the advent of the use of personality traits in the DSM-5 (American Psychiatric Association, 2013). Many psychologists today agree that the FFM framework can be used as a foundation for integrating common and abnormal personality traits (Markon, Krueger, & Watson, 2005). The usefulness of FFM has received much empirical support (see a review by Miller, 2012).

## The Lower Order Facet Structure of the FFM

Organizing personality into five trait domains is often too general for certain purposes, such as when differentiating job candidates for a specific task or individualizing clinical diagnoses. For instance, recognizing that someone is high on trait factor Extraversion could indicate that the person is sociable, happy, energetic, or dominant, or all of these. In other words, what is the scope and meaning when someone scores high on Extraversion? Another example might be illustrated by the trait factor Openness. There are several specific lower order facet traits which could be more informative, such as Adventurousness in predicting tendency to travel, or Intellect in predicting choice of education. Research and practice might therefore be better served by using *narrower and more specific* traits. Facets should enable higher precision of analysis (see Ziegler & Bäckström, 2016). The IPIP-NEO instrument makes use of this by including a number of facet traits, consisting of dispositions towards certain behaviors, affects, and cognitions within each factor domain (see Zillig, Hemenover, & Dienstbier, 2002). The 30 facet traits of IPIP-NEO are presented in Table 1, with 6 facet traits for each one of the 5 trait domains, arranged in columns, followed by example items. For instance, the first facet trait Friendliness in trait domain Extraversion (E1) attempts to capture the propensity of being friendly and experienced as socially warm person. One item example of the Friendliness facet trait is the extent one “makes friends easily”. Personality models are often constructed by items making up facet traits which in turn make up the broad trait domain, which aims at generating both content scope and precision (Johnson, 2014; McCrae, 2010). The extent to which this hierarchical structure is valid can be tested by assessing how items, facets, and trait factor are interlinked. Optimally, both trait and facet levels should be saturated by the variance provided by items.

**Table 1:** The Five Personality Traits and their Underlying Facet Traits in the IPIP-NEO-120

Neuroticism	Extraversion	Openness	Agreeableness	Conscientiousness
N1_Anxiety *Worry about things*	E1_Friendliness *Make friends easily*	O1_Imagination *Love to daydream*	A1_Trust *Trust others*	C1_Self-efficacy *Excel in what I do*
N2_Anger *Get angry easily*	E2_Gregarious *Love large parties*	O2_Artistic *Believe in the importance of art*	A2_Morality *Cheat to get ahead*	C2_Orderliness *Like to tidy up*
N3_Depression *Often feel blue*	E3_Assertive *Take charge*	O3_Emotionality *Feel other’s emotions*	A3_Altruism *Love to help others*	C3_Dutifulness *Keep my promises*
N4_Self-conscious *Find it difficult to approach others*	E4_Activity *Am always busy*	O4_Adventurous *Prefer variety to routine*	A4_Cooperation *Love a good fight*	C4_Achievement *Work hard*
N5_Immoderation *Rarely overindulge*	E5_Excitement *Love excitement*	O5_Intellect *Love to read challenging material*	A5_Modesty *Think highly of myself*	C5_Self-discipline *Am always prepared*
N6_ Vulnerability *Panic easily*	E6_Cheerful *Radiate joy*	O6_Liberalism *Tend to vote for liberal political candidates*	A6_Sympathy *Sympathise with the homeless*	C6_Cautiousness *Rush into things*

*Note.* Each one of the 30 facet traits in IPIP-NEO are exemplified with a typical item worded in *italics*.

## The Present Study

The overall endeavor of the present study was to test the structure of one public domain version of the Five Factor Model (FFM) of personality, IPIP-NEO-120 (Johnson, 2014). The specific objective was to investigate how facet traits organize according to its proposed structure (Table 1) in respective FFM trait domain. The general hypothesis was that facet traits would contribute to each trait domain according to their positions in the established FFM literature (Costa & McCrae, 1995). Confirming the structure of personality assessment in the public domain would give important information on scope and meaning of this publically available FFM to professionals who need a simple and accessible instrument in their research and practice.

## Method

### Sample and Procedure

We were able to make use of one of the largest US samples to date (*N* = 320,128). The sample consisted of 40% males (*N* = 127,695) and 60% females (*N* = 192,433), with an average age of 28.1 years (*SD* = 10.1). The respondents were 19–69 year old. The data was collected through an online survey, in the form of a website offering visitors feedback on their FFM personality after filling out 120 items. The visitors were at large volunteers from all walks of life, nationwide in the US, who could have reached the site by word-of-mouth, Internet search engines, and other informal channels. Every participant had to actively acknowledge that the survey would be time-consuming, used for educational and research purposes, and that careless responding would invalidate the usefulness. No login was required, and no personal, traceable data (e.g., IP-addresses) was collected, neither data on ethnicity, socio-economic status, or other demographic characteristics. The data-set can be accessed at an open psychology research depository (see Supplementary Materials).

### Data Limitations

Despite the generous number of respondents this likely is not a representative nationwide sample. Due to the active volunteering of respondents, a reasonable assumption is that several of the trait facet levels such as in Openness to Emotionality, Openness to Intellect, and Altruism were likely unrepresentatively high (see Appendix), since these traits are known to characterize people interested in psychology (Vedel, 2016).

Another concern was that online surveys are often known for inconsistent responding or intentional misrepresentations. However, the quality of the present online survey data was analyzed according to guidelines in Johnson (2005). For example, duplicates (showing long strings of equivalent data) were removed. Also, repetitive patterns, which may indicate curiosity to see the items with no purpose of doing going through with the test, were removed. The missing data (1%) was finally corrected by imputing item means. Due to the present size of sample (*N* = 320,128), the item-correlations with and without the missing data imputed showed near perfect convergence (*r* = .998). The conclusion is that these commonly raised problems may not interfere with the purpose of the present study.

### Instrument

IPIP-NEO-120 is a publically available representation of the FFM (Johnson, 2014), drawing 120 items from the International Personality Item Pool (IPIP; Goldberg et al., 2006). IPIP-NEO was built on open-source items correlating with the original NEO-PI-R (Costa & McCrae, 1995). IPIP-NEO was created seeking to optimize length, reliability, and validity in FFM measurement, and even surpassed the original in mean facet reliability (α > .80) (Johnson, 2014). The trait scales are built by four correlated items within each facet trait, while keeping bandwidth in meaning (i.e., removing repetitive items), as well as tending to balancing items (+ and – keyed items). Each of the items are measured on a 1 (almost never) – 5 (almost always) scale which then are summarized into facet traits (Min = 4, Max = 20). Six facet traits in turn are averaged into one trait factor. Overall, the mean reliability for facet scales in the present study was α = .78. Only four facets (13%) were below α = .70. See Table 2 for a summary of alphas.

**Table 2:** Exploratory Factor Analysis of IPIP-NEO Facet Traits

Trait domain / facet trait	α	C	E	A	N	O
Conscientiousness	.90					
C1_Self-efficacy	.77	**.74**	.04	-.09	-.11	.08
C2_Orderliness	.85	**.49**	.02	.10	.04	-.21
C3_Dutifulness	.67	**.46**	-.06	.44	-.03	-.14
C4_Achievement	.78	**.76**	-.04	.10	.08	.08
C5_Self-discipline	.72	**.80**	.05	.02	-.05	-.09
C6_Cautiousness	.88	**.38**	-.26	.31	-.26	-.12
Extraversion	.89					
E1_Friendliness	.81	.08	**.85**	.11	-.06	-.07
E2_Gregariousness	.80	-.02	**.83**	-.08	.03	-.04
E3_Assertiveness	.86	***.53***	**.25**	-.33	-.07	.16
E4_Activity	.71	***.55***	**.19**	-.08	.14	.02
E5_Excitement	.74	-.17	**.45**	-.39	-.01	.25
E6_Cheerfulness	.80	.11	**.61**	.14	-.18	.01
Agreeableness	.85					
A1_Trust	.86	-.09	***.40***	**.38**	-.13	-.05
A2_Morality	.74	.20	-.07	**.62**	-.07	.05
A3_Altruism	.73	.11	.31	**.57**	.23	.27
A4_Cooperation	.69	-.01	-.05	**.72**	-.22	-.03
A5_Modesty	.72	-.19	-.13	**.46**	.18	-.05
A6_Sympathy	.73	.01	.16	**.52**	.22	.31
Neuroticism	.90					
N1_Anxiety	.79	.00	-.13	-.02	**.83**	-.07
N2_Anger	.87	.12	-.09	-.45	**.55**	.00
N3_Depression	.85	-.21	-.39	-.05	**.47**	.14
N4_Self-conscious	.72	-.11	-.64	.14	**.22**	-.06
N5_Immoderation	.72	-.29	.09	-.23	**.27**	.16
N6_Vulnerability	.78	-.17	-.09	.03	**.76**	-.10
Openness	.82					
O1_Imagination	.75	-.21	-.02	-.14	.07	**.52**
O2_Artistic	.75	.02	-.06	.19	-.02	**.64**
O3_Emotionality	.66	.08	.17	.32	***.45***	**.37**
O4_Adventurous	.71	-.05	.18	-.04	-.35	**.45**
O5_Intellect	.74	.12	-.23	-.02	-.26	**.64**
O6_Liberalism	.69	-.19	-.04	.02	-.01	**.32**

*Note*. Boldface italics indicates factor loading not aligning to expectation (i.e., higher loading on the wrong trait factor). 95% CI were less than ±0.01.

### Statistical Method

The analyses were based on the 30 facet traits in the FFM (IPIP-NEO-120). We first explored the unconstrained factor structure with facet correlations and exploratory factor analysis to see if facets organized into five proposed factors. The main objective was then finalized with testing hierarchical structural CFA models, one for each trait domain. The models were based on established FFM literature (Costa & McCrae, 1995), and tested the loadings between manifest items, latent trait facets, and respective latent trait domain. We utilized fit indices for two ways of modeling each FFM trait domain. The first way was the original and most used hierarchical second-order structure in two levels (Costa & McCrae, 1995). This is characterized by the general latent trait domain at the top, loaded by six facet traits, which in turn are loaded by 24 item measurement items. The second way of modeling was a bi-factor model, where both the general latent trait factor and specific latent facets are loaded onto directly by measurement items. The bi-factor approach is also helpful in revealing if any facet traits could be viewed as trait domains on their own—If items would not load on the general trait domain factor, but only on the facet trait, this could imply independence from the FFM domains. The CFA model was conducted in a SEM-framework, extracted with Maximum Likelihood, using AMOS v.23. The models were attempted as largely unconstrained, without covarying error terms. Due to the large sample size, all results were significant and standard errors were less than 0.01.

## Results

**Figure 1:**
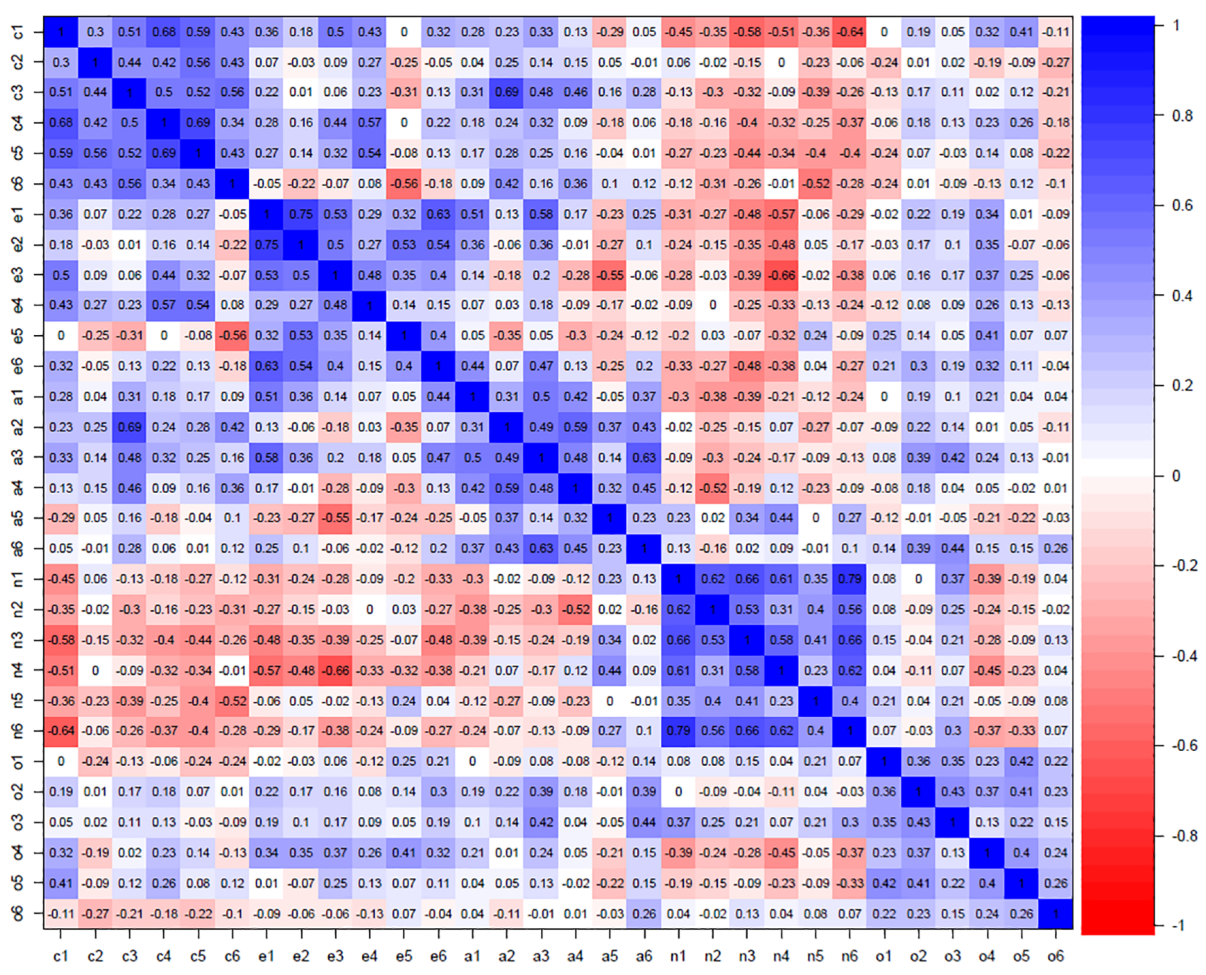
Correlations between the 30 facet traits in the Five Factor Model IPIP-NEO-120 (*N* = 320,128). *Note*. The more intense blue color, the higher positive correlation, the more intense red color, the higher negative correlation. C1-C6 = Conscientiousness facets; E1-E6 = Extraversion facets; A1-A6 = Agreeableness facets; N1-N6 = Neuroticism facets; O1-O6 = Openness facets. Descriptions of facet abbreviations are found in Table 1.

Figure 1 presents all 30 facet correlations in IPIP-NEO (red colors showing negative relationships and blue colors showing positive relationships).

Overall, the contrasting intensity of blue along the diagonal generally shows a high internal coherence within the five trait domains. Equally, the rows and columns of the Neuroticism factor (facets n1-n6) show intensity of red colors, indicating overall negative correlations with the other four trait domains. Apart from these patterns, most facets showed weak or no color with cross-facet traits, indicating fair, but far from perfect, discriminant qualities.

### Exploring Five Factors in the IPIP-NEO

Before investigating the main objective of testing the five-factor structure as measured by IPIP-NEO, we checked whether Exploratory Factor Analysis (EFA) would confirm five factors. Utilizing Maximum Likelihood (ML) for extraction, and oblique rotation, assuming correlations between facets, the summary of loadings (structural pattern) is reported in Table 2. Only five factors had Eigenvalues above 1, which was an initial confirmation of the FFM IPIP-NEO structure. The goodness of fit was χ^2^ (295) = 38,877, RMSEA = 0.07, and TLI = 0.84. The first factor extracted 21%, the second 11%, the third 8%, the fourth 6%, and the fifth 5% of total variance, making the total 51%. Overall, the facet traits revealed five clear trait structures (Table 2). However, there were also 4 facet discrepancies, as marked in *italics* in Table 2. Facet Trust (A1) loaded slightly more on Extraversion; Assertiveness (E3) and Activity (E4) organized mostly under Conscientiousness; and Emotionality (O3) under Neuroticism.

#### The Structure of the Five Factors in the IPIP-NEO

**Figure 2:**
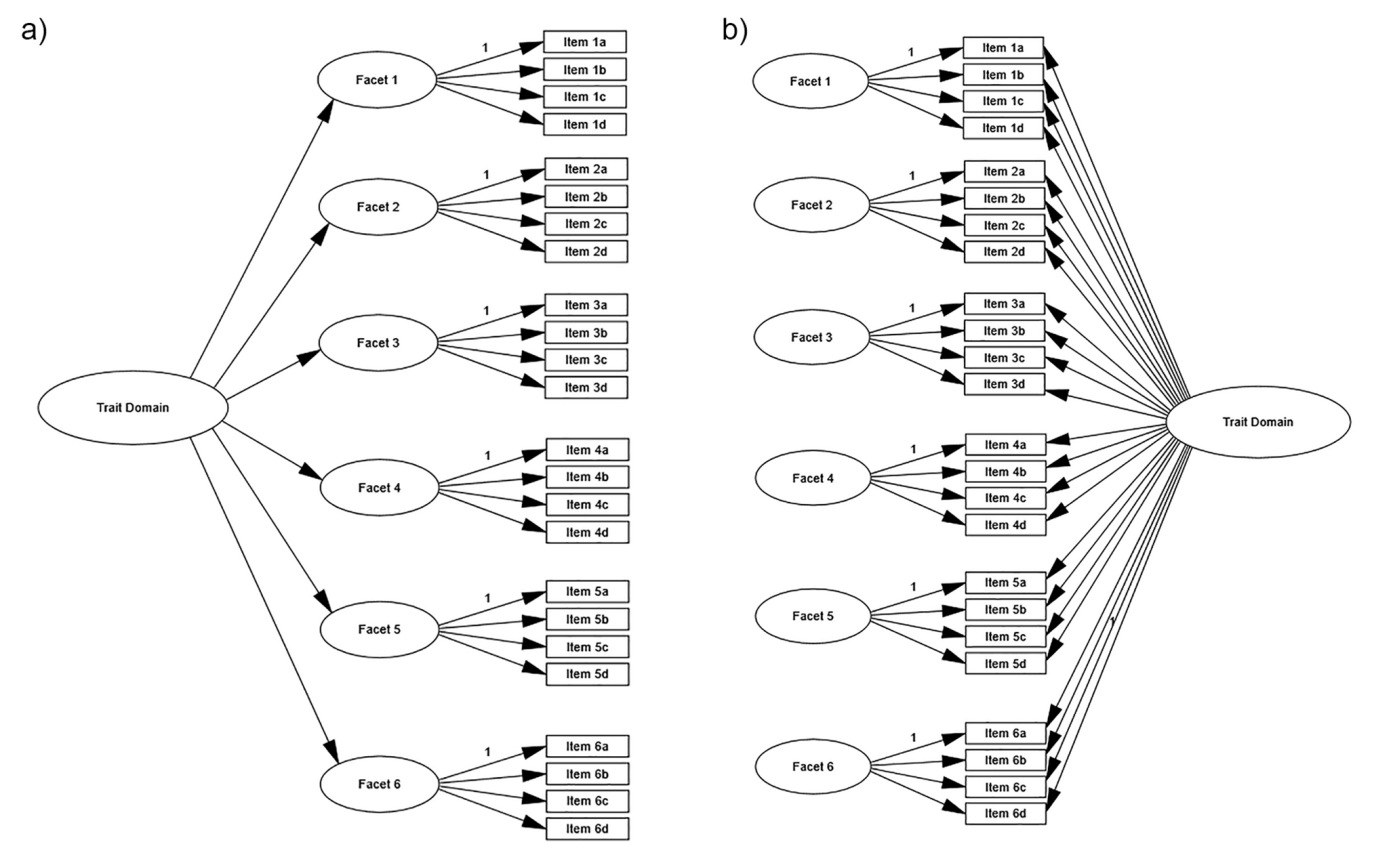
a) The structure of the FFM second-order models. b) The structure of the FFM bi-factor models. *Note.* FFM = Five Factor Model.

The main objective was to test the structures of the FFM trait domains in the public IPIP-NEO-120 instrument. All five CFA trait structure models, one for each domain, were conducted in two ways: Second-order models; See Figure 2a, and bi-factor models; See Figure 2b). All parameters were unconstrained, and conducted without modifications (e.g., covariances between errors).

Table 3 accounts for the results of the second-order models (based on Figure 2a), one for each FFM domain. The table includes fit indices (χ^2^(*df*), RMSEA, TLI, and CFI), reported in the trait domain rows, and facet and item loadings, reported in the facet rows. The first observation was that the second-order structures overall showed at best tolerable model fits. Most were above recommended stricter limits (RMSEA > 0.05 and CFI, TLI < 0.90; Cheung & Rensvold, 2002). Fit indices, however, could be improved by allowing one or two selected error variances to covary within each trait facet, which would result in Neuroticism, Openness, and Conscientiousness structures fitting within recommended limits (RMSEA < 0.05 and CFI, TLI > 0.90). A second observation was that loadings both on facet and trait factor level were overall high. The average facet loading in the second-order models was 0.65, and the average item loading 0.70. However, a few weaker loadings (defined as β < .50) were seen, such as facet traits Trust and notably Modesty in the Agreeableness domain, or Assertiveness and Activity in the Extraversion domain. Openness showed the overall weakest construct coherence, with three of its facet traits, that is, Imagination, Emotionality, and Liberalism, showing weak loadings. Such facet traits may not be contributing robustly to their respective general trait factors. Also, on the contrary, several facet traits showed notably strong loadings (β > .90)—Friendliness on Extraversion, Self-discipline on Conscientiousness, and Anxiety and Vulnerability on Neuroticism almost showed convergent identity.

**Table 3:** IPIP-NEO Second-order FFM Trait Structures (Based on Figure 2a)

Trait domain / facet trait	ECV	Facet (β)	Item a	Item b	Item c	Item d
Neuroticism χ^2^(246) = 23,295; RMSEA = 0.06; TLI = 0.90, CFI = 0.91						
N1_Anxiety	.49	1.0^a^	.62	.67	.68	.79
N2_Anger	.62	.57	.85	.82	.82	.65
N3_Depression	.59	.72	.82	.73	.87	.65
N4_Self-conscious	.40	.54	.74	.61	.63	.53
N5_Immoderation	.40	.41	.61	.62	.61	.68
N6_Vulnerability	.47	.98	.77	.70	.64	.62
Extraversion χ^2^(246) = 30,751; RMSEA = 0.07; TLI = 0.87, CFI = 0.88						
E1_Friendliness	.53	.96	.72	.79	.75	.63
E2_ Gregarious	.51	.89	.72	.76	.60	.75
E3_Assertive	.61	.47	.85	.76	.81	.70
E4_Activity	.44	.43	.77	.83	.60	.29
E5_Excitement	.42	.54	.73	.73	.49	.60
E6_Cheerfulness	.51	.75	.66	.74	.73	.72
Openness χ^2^(246) = 17,301; RMSEA = 0.05; TLI = 0.88, CFI = 0.89						
O1_Imagination	.45	.45	.52	.63	.79	.71
O2_Artistic	.44	.88	.77	.55	.60	.70
O3_Emotionality	.39	.41	.54	.59	.50	.66
O4_Adventurous	.33	.46	.52	.68	.69	.59
O5_Intellect	.44	.67	.54	.73	.59	.77
O6_Liberalism	.43	.35	.88	.34	.83	.35
Agreeableness χ^2^(246) = 26,313; RMSEA = 0.06; TLI = 0.86, CFI = 0.87						
A1_Trust	.61	.42	.81	.69	.81	.81
A2_Morality	.43	.63	.68	.59	.83	.49
A3_Altruism	.41	.91	.63	.74	.62	.57
A4_Cooperation	.37	.64	.49	.58	.72	.63
A5_Modesty	.47	.13	.46	.87	.92	.24
A6_Sympathy	.42	.78	.66	.71	.57	.64
Conscientiousness χ^2^(247)* = 17,513; RMSEA = 0.053; TLI = 0.92, CFI = 0.93						
C1_Self-efficacy	.46	.89	.73	.65	.67	.72
C2_Orderliness	.58	.55	.69	.70	.84	.80
C3_Dutifulness	.42	.62	.80	.57	.35	.79
C4_Achievement	.49	.81	.72	.69	.75	.63
C5_Self-discipline	.40	.99	.99	.67	.66	.45
C6_Cautiousness	.66	.49	.82	.77	.83	.82

*Note*. Each FFM facet loads (β) on the top trait domain factor, and is made up by four items (a, b, c, and d). See Figure 2a. ECV = Explained Common Variance (based on respective items); FFM = Five Factor Model; RMSEA = Root Mean Square Error of Approximation; TLI = Tucker Lewis Index; CFI = Comparative Fit Index. 95% CI were less than ±0.01.

^a^A standardized beta equal to or above 1.0 can legitimately occur, and indicates high multicollinearity (Deegan, 1978).

Table 4 accounts for the bi-factor models (based on Figure 2), one for each FFM domain. The first observation here was that the bi-factor models, which control for a general trait factor, overall showed better model fits than second-order models. This is however expected since fit indices such as RMSEA reward parsimony (i.e., adding constraints). Nevertheless, CFI were also superior, and the difference was substantial for two of the trait structures: Extraversion (.90 compared to .87) and Agreeableness (.91 compared to .86). A second result was that ECV average was .47 on facet level and .50 on trait factor level. These results can overall be interpreted as unidimensionality in the components of the IPIP-NEO structure, which support a more nuanced facet structures as hypothesized. Along this line of reasoning, however, some trait structures had facets that seemed disconnected from the trait domain. For instance, the trait factor Agreeableness and facet Modesty showed a disconnection. Items “I think I am better than others” and “I think highly of myself” did not load on the general trait domain Agreeableness (β = .05 and β = .09), but highly on the facet trait (β = .88 and β = .90). (Recall that facet trait Modesty did not load satisfactorily on Agreeableness in the second-order model). Also, Self-discipline (in trait domain Conscientiousness) and Friendliness (in Extraversion) items even loaded negatively on respective facets, while loading strongly on respective general trait factors. This could be interpreted as these facets being identical with the general trait factor.^i^ These mentioned examples may represent the core of respective general factors but may at the same time be too similar to optimally function as independent facet traits.

**Table 4:** IPIP-NEO Bi-factor FFM Trait Structures (Based on Figure 2b)

Trait domain / facet trait	Item a (G)	Item b (G)	Item c (G)	Item d (G)
**Neuroticism χ^2^(230)^a^ = 20,704; RMSEA = 0.06; TLI = 0.90, CFI = 0.92, ECV = 0.52**				
N1_Anxiety	.*16* (.60)	.57 (.65)	.*13* (.66)	.55 (.80)
N2_Anger	.75 (.44)	.61 (.52)	.71 (.44)	.49 (.41)
N3_Depression	.58 (.58)	.46 (.54)	.66 (.60)	.37 (.51)
N4_Self-conscious	.66 (.38)	.50 (.34)	.53 (.34)	.38 (.35)
N5_Immoderation	.54 (.28)	.63 (.*18*)	.56 (.24)	.58 (.33)
N6_Vulnerability	.56 (.76)	.31 (.69)	.*10* (.63)	.*04* (.62)
**Extraversion χ^2^(228) = 22,111; RMSEA = 0.06; TLI = 0.90, CFI = 0.91, ECV = 0.55**				
E1_Friendliness	-.*01* (.73)	.*09* (.76)	.64 (.71)	.*12* (.62)
E2_Gregarious	.70 (.58)	.28 (.69)	.*10* (.58)	.28 (.66)
E3_Assertive	.77 (.38)	.65 (.37)	.73 (.34)	.59 (.37)
E4_Activity	.79 (.25)	.70 (.39)	.49 (.35)	.31 (.*03*)
E5_Excitement	.45 (.46)	.48 (.42)	.71 (.06)	.63 (.32)
E6_Cheerfulness	.32 (.55)	.31 (.64)	.60 (.50)	.62 (.49)
**Openness χ^2^(228) = 12,405; RMSEA = 0.05; TLI = 0.91, CFI = 0.92, ECV = 0.43**				
O1_Imagination	.31 (.46)	.49 (.35)	.84 (.27)	.59 (.35)
O2_Artistic	.39 (.64)	.*11* (.56)	.29 (.51)	.59 (.53)
O3_Emotionality	.47 (.25)	.51 (.29)	.42 (.25)	.69 (.*18*)
O4_Adventurous	.44 (.27)	.57 (.35)	.70 (.26)	.45 (.36)
O5_Intellect	.25 (.48)	.60 (.46)	.33 (.47)	.63 (.48)
O6_Liberalism	.80 (.31)	.30 (.*16*)	.82 (.25)	.31 (.17)
**Agreeableness χ^2^(228) = 15,003; RMSEA = 0.05; TLI = 0.91, CFI = 0.93, EVC = 0.48**				
A1_Trust	.76 (.30)	.57 (.39)	.77 (.28)	.71 (.38)
A2_Morality	.49 (.48)	.37 (.46)	.59 (.60)	*.17* (.48)
A3_Altruism	.40 (.49)	.62 (.55)	.22 (.56)	.24 (.50)
A4_Cooperation	.39 (.33)	.58 (.35)	.40 (.52)	.33 (.52)
A5_Modesty	.41 (.45)	.88 (.*05*)	.90 (.*09*)	.20 (.35)
A6_Sympathy	.68 (.37)	.54 (.46)	.*19* (.52)	.41 (.47)
**Conscientious χ^2^(228) = 13,700; RMSEA = 0.05; TLI = 0.94, CFI = 0.95, ECV = 0.53**				
C1_Self-efficacy	.23 (.66)	.38 (.54)	.37 (.57)	.42 (.56)
C2_Orderliness	.57 (.40)	.54 (.43)	.72 (.45)	.66 (.45)
C3_Dutifulness	.66 (.47)	.38 (.40)	.*13* (.34)	.64 (.47)
C4_Achievement	.35 (.60)	.38 (.56)	.52 (.59)	.40 (.50)
C5_Self-discipline	-.*13* (.64)	-.*05* (.66)	.39 (.66)	.29 (.56)
C6_Cautiousness	.72 (.39)	.66 (.39)	.73 (.40)	.69 (.45)

*Note*. FFM = Five Factor Model; RMSEA = Root Mean Square Error of Approximation; TLI = Tucker Lewis Index ; CFI = Comparative Fit Index. Each FFM facet is made up by four items (a, b, c, and d), and is controlled by G (General trait domain factor). See Figure 2b. Very weak loadings (below .20) are marked in *italics*. 95% CI were less than ±0.01. Fit indices (χ^2^, RMSEA, TLI, CFI) are reported based on Figure 2b, without improvement modifications (e.g., covariations between errors or between facets). ^a^Did not converge in a first run with all parameters free, returning negative error variance or Heywood cases, which was amended by relaxing two constraints.

A last and final complexity was that all the trait domain models may be confounded by common variance bias, such as social desirability or acquiescence responding. This could potentially confound fit indices, as well as loadings (See the extended discussion in Anusic, Schimmack, Pinkus, & Lockwood, 2009). Consequently, we also attempted to estimate to what extent a common method factor may be present. We created a so called bogus marker variable, which was made up by four uncorrelated fake items, which we added to the bi-factor model of the trait structure Agreeableness, chosen for its face appeal and social desirability. The average loadings from this fictive variable on the common factor, even though small, were not trivial (β = .18). With no common method variance present, this bogus variable should have been closer to zero. This result indicated that almost 4% of the variance in Agreeableness may be attributed to common method factors due to the respondent source.

## Discussion

We were able to confirm the established five factor structure in the public-domain version of the FFM, IPIP-NEO-120, in one of the largest US public samples to date. The inter-correlation matrix (Figure 1), as well as the exploratory factor analysis (Table 2) reported five clearly recognizable factor patterns. The main objective of testing hierarchical second-order models and bi-factor CFA models (Figures 2a and b) overall reported tolerable fit indices, and mostly sufficient structural loadings (Tables 3 and 4). However, these were at times not overly impressive. The results may be interpreted in a more optimistic light, seeing the often notoriously low model fit indices and weak model structures in complex personality models (Hopwood & Donnellan, 2010). It was clear that the five trait factors were supported by a substructure made up of facet traits, based on the ECV-values (≈ .50; see Table 4). The ECV indicated that substantial common variance is present in the IPIP-NEO-items, assuming trait unidimensionality and thus supporting a more nuanced facet structure. Only trait factor Openness had a relatively low ECV of .43 and is the one factor in the IPIP-NEO that is more loosely structured, being composed of items constituting various facets such as Imagination, Liberalism, and Intellect (see Table 1). The IPIP-NEO-120 results encourage careful future use of the five trait domains and 30 facet traits in the public domain. This use is presently ongoing, such as being used in subclinical contexts (Kajonius, 2017), or nation-wide comparisons (Kajonius & Mac Giolla, 2017; Mac Giolla & Kajonius, 2018).

In line with previous research, the present study also identified some overall concerns regarding trait domain validity in the IPIP-NEO-120. Despite the FFM being an empirically impressive model, many critics have pointed out apparent limitations, such as lack of robust scope and meaning (see McCrae, 2010). One measurement objective is that facet traits should help define the broader trait domains, not confuse these. There may be both independent facet traits (e.g., Modesty) as well as perhaps domain-convergent facet traits (e.g., Self-discipline and Friendliness) in each of the FFM trait domains, as the present study indicates. The IPIP-NEO has undoubtedly inherited some problems from the original NEO-PI-R model (cf. Costa & McCrae, 1992; Furnham, Guenole, Levine, & Chamorro-Premuzic, 2013). One example in the present study is that facet traits Openness to Imagination, Emotionality, and Liberalism are weakly (β < .50) related to the general Openness domain. Another example is the Activity and Assertiveness facets in Extraversion. In the IPIP-NEO, Openness seems to be more characterized by artistic (esthetic) interests and intellectual endeavors, rather than emotions and politics (see facet loadings in Table 3), and Extraversion seems better characterized by social energy and positive temperaments, than being busy and assertive (which tended to sort under Conscientiousness, when unconstrained in EFA; see Table 1).

Moreover, one specific finding worth highlighting is that the facet trait Modesty was almost entirely disconnected from trait domain Agreeableness, and may surprisingly be the one facet currently not functioning sufficiently. One explanation could be that Modesty should indeed be viewed as an independent trait apart from Agreeableness. Modesty may be more linked to a proposed sixth factor in an expanded FFM paradigm, Honesty-Humility (cf. Kajonius & Dåderman, 2014). Another explanation may be that Modesty contains a greater degree of social desirability than other facet traits (Allik et al., 2010). Not many would score an item such as “I think I am better than others” highly, and this may not provide enough variance to support a general Agreeableness trait. This anomaly may again fuel the debate concerning a sixth factor in the FFM, suggesting the addition of Honesty-Humility (e.g., DeYoung, Quilty, & Peterson, 2007). Such a factor may capture additional normative values, beyond Agreeableness. Either way, reporting the contents and structural validity of a trait domain is a crucial key factor for understanding what is being measured.

The other side of the coin is that a few IPIP-NEO facet traits also showed, not weak or disorganized loadings, but extremely strong loadings bordering on identity. Facet traits Self-discipline under Conscientiousness, Friendliness under Extraversion, and Anxiety under Neuroticism reported CFA loadings around and above β = .90. This may be interpreted as the facet trait being identical to the trait domain, and could be considered too convergent for a multifaceted structure. Conversely, this can also be considered as core features of respective FFM trait domains, and can be helpful when creating and choosing items for shorter personality measurements with fewer items.

It is clear that there remains a challenge on how to establish conceptualizations of personality trait domains—Are they mostly descriptions of underlying temperaments, or behavioral life-styles, thus yielding different scopes and meanings to the trait domains (cf. Zillig et al., 2002)? The present structural analyses, using one of the largest population samples to date, may facilitate this discussion by reporting overall results pertaining to what is being measured in each of the five trait domains. (For instance, IPIP-NEO Openness measures mostly intellectual/artistic curiosity, and Extraversion measures mostly being happy in the fellowship of other people). The complexity and hierarchy of psychological personality traits may ultimately never be captured in a simple structural model (Hopwood & Donnellan, 2010). Nevertheless, the present IPIP-NEO seems to be reasonably robust compromise. The take-home message from the present study is the demonstrated structure of lower order FFM facet traits, which provides some of the needed scope and precision for future needs of measurements of individual differences.

## Data Availability

Raw data collected with both a 300-item and 120-item IPIP version of the NEO PI are freely available (see the Supplementary Materials section).

## Notes

Raw data collected with both a 300-item and 120-item IPIP version of the NEO PI (for access see Index of Supplementary Materials below).

## Appendix

**Table A1:** Sample Descriptive Statistics of 5 Trait Domains and 30 Facets

Trait domain / facet trait	*M*	*SD*	α
Neuroticism	11.10	2.66	0.90
N1_Anxiety	12.07	3.77	0.79
N2_Anger	11.51	4.11	0.87
N3_Depression	9.35	3.87	0.85
N4_Self-conscious	11.69	3.64	0.72
N5_Immoderation	11.94	3.44	0.72
N6_Vulnerability	10.07	3.63	0.78
Extraversion	13.69	2.36	0.89
E1_Friendliness	14.48	3.60	0.81
E2_Gregarious	12.36	4.02	0.80
E3_Assertive	14.56	3.43	0.86
E4_Activity	12.84	3.15	0.71
E5_Excitement	12.52	3.32	0.74
E6_Cheerfulness	15.35	3.20	0.80
Openness	13.71	2.06	0.82
O1_Imagination	14.60	3.41	0.75
O2_Artistic	14.67	3.58	0.75
O3_Emotionality	15.20	3.01	0.66
O4_Adventurous	12.28	3.26	0.71
O5_Intellect	14.50	3.55	0.74
O6_Liberalism	11.03	3.67	0.69
**Agreeableness**	14.87	2.01	0.85
A1_Trust	13.43	3.54	0.86
A2_Morality	16.63	2.88	0.74
A3_Altruism	16.72	2.56	0.73
A4_Cooperation	15.10	3.49	0.69
A5_Modesty	12.34	3.35	0.72
A6_Sympathy	15.03	3.10	0.73
**Conscientiousness**	14.95	2.34	0.90
C1_Self-efficacy	16.32	2.40	0.77
C2_Orderliness	13.23	4.31	0.85
C3_Dutifulness	16.32	2.57	0.67
C4_Achievement	16.06	3.09	0.78
C5_Self-discipline	14.07	3.16	0.72
C6_Cautiousness	13.68	4.09	0.88

*Note. N* = 320,128. *SD* = standard deviation. The facet trait scales ranged from 4 (Min) to 20 (Max). *N*_Male_ = 127,695, *N*_Female_ = 192,433, *M*_Age_ = 28.13, *SD* = 10.14. 95% CI were ±0.01.
